# Uric Acid Is More Strongly Associated with Impaired Glucose Regulation in Women than in Men from the General Population: The KORA F4-Study

**DOI:** 10.1371/journal.pone.0037180

**Published:** 2012-05-16

**Authors:** Christa Meisinger, Angela Döring, Doris Stöckl, Barbara Thorand, Bernd Kowall, Wolfgang Rathmann

**Affiliations:** 1 Helmholtz Zentrum München, German Research Center for Environmental Health (GmbH), Institute of Epidemiology II, Neuherberg, Germany; 2 MONICA/KORA Myocardial Infarction Registry, Central Hospital of Augsburg, Augsburg, Germany; 3 Helmholtz Zentrum München, German Research Center for Environmental Health (GmbH), Institute of Epidemiology I, Neuherberg, Germany; 4 Department of Obstetrics and Gynaecology, Campus Grosshadern, Ludwig-Maximilians-University, Munich, Germany; 5 Institute of Biometrics and Epidemiology, German Diabetes Center, Leibniz Center for Diabetes Research at Heinrich Heine University, Düsseldorf, Germany; John Hopkins Bloomberg School of Public Health, United States of America

## Abstract

**Objective:**

High serum uric acid (UA) levels are associated with the metabolic syndrome, type 2 diabetes and cardiovascular disease. It is largely unknown whether there are gender-specific differences regarding the association between UA and prediabetic states. We examined the possible association between UA levels and known as well as newly diagnosed diabetes (NDD), isolated impaired fasting glucose (i-IFG), isolated impaired glucose tolerance (i-IGT), and combined IFG/IGT in a population-based sample of 32-to-81-year-old men and women.

**Research Design and Methods:**

An oral glucose tolerance test was carried out in all 2,740 participants without known diabetes of the Cooperative Health Research in the Region of Augsburg (KORA) F4 Study conducted between 2006 and 2008 in Southern Germany. Serum UA was analysed by the uricase method.

**Results:**

In women after multivariable adjustment the associations between UA and i-IFG (OR 1.57, 95% CI 1.15–2.14), IFG/IGT (OR 1.52, 1.07–2.16), NDD (OR 1.67, 95% CI 1.28–2.17), and known diabetes (OR 1.47, 95% CI 1.18–1.82) remained significant, but the association with i-IGT (OR 1.14, 95% CI 0.95–1.36) lost significance. In contrast in men, after multivariable adjustment there was only a significant association between UA levels and i-IFG (OR 1.49, 95% CI 1.21–1.84), all other associations were non-significant (i-IGT: OR 1.09, IFG/IGT: OR 1.06, NDD: OR 0.91, known diabetes: OR 1.04; all p-values>0.05).

**Conclusions:**

Serum UA concentrations were associated with different categories of impaired glucose regulation in individuals from the general population, particularly in women. Further studies investigating the role of UA in the development of derangements in glucose metabolism are needed.

## Introduction

Uric acid is the final oxidation product of purine metabolism in humans. There is evidence that hyperuricemia is associated with the metabolic syndrome [1], [2] and incident type 2 diabetes [3], [4]. While some investigations found no association between uric acid levels and the risk of diabetes mellitus [5], in a recent meta-analysis a positive association was reported [6]. Although uric acid levels are substantially different between men and women [7], most of the prior studies on this issue were conducted in male populations alone and, if including both men and women, did not conduct gender specific analyses [8]–[10]. Thus, it remains largely unknown whether there are gender differences regarding the relationship between uric acid levels and impaired glucose regulation.

In addition, so far, population-based data to determine whether uric acid levels are associated with different stages of impaired glucose regulation, in particular prediabetic states, is scarce [11]–[13]. Therefore, the present study was set out to investigate the possible association between uric acid levels and known diabetes, newly diagnosed diabetes (NDD), isolated impaired fasting glucose (i-IFG), isolated impaired glucose tolerance (i-IGT), and combined IFG/IGT in a population of 32-to-81-year-old men and women in Southern Germany. Given the marked differences in serum uric acid levels between men and women, gender-specific analysis was performed.

## Methods

**Figure 1:**
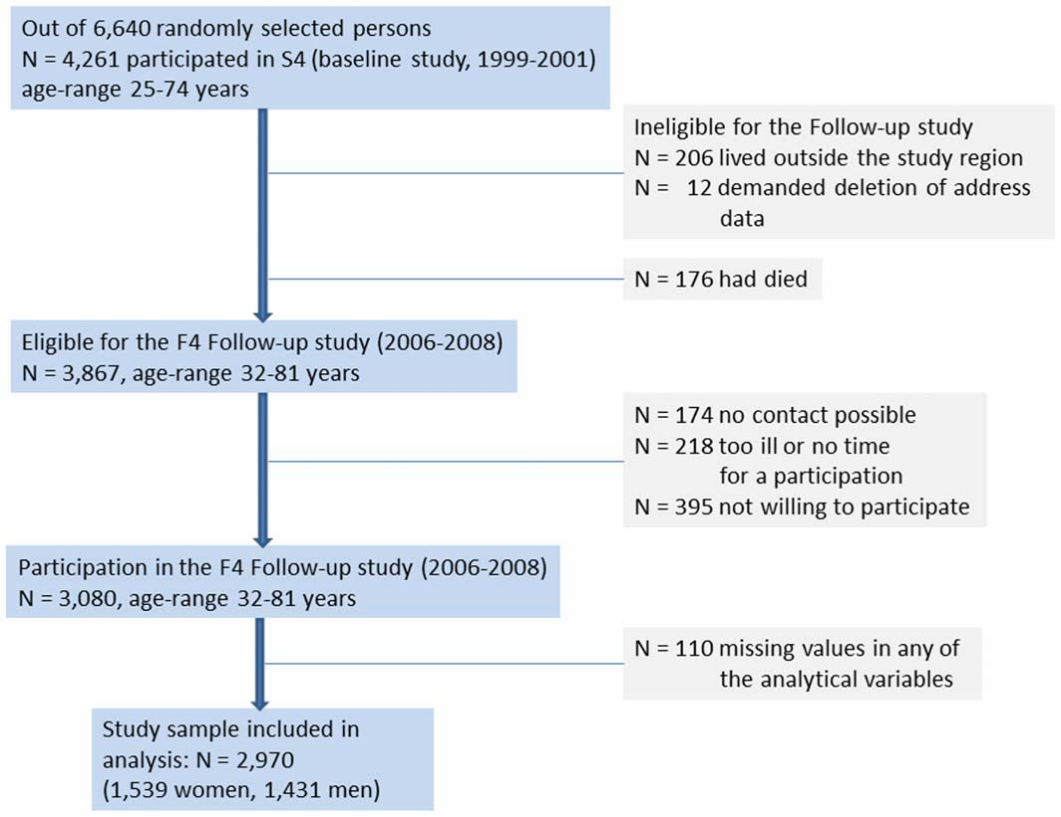
Flow chart describing the selection and subsequent loss of participants.

Data are based on the Cooperative Health Research in the Region of Augsburg (KORA) F4 study (2006–2008), a follow-up of the KORA S4 study, a population-based health survey conducted in the city of Augsburg and two surrounding counties between 1999 and 2001. For S4 a total sample of 6,640 subjects was drawn from the target population consisting of all German residents of the region aged 25 to 74 years. Of all 4,261 participants of the S4 baseline study, 3,080 also participated in the 7-year follow-up F4 study. Persons were considered ineligible for F4 if they lived outside the study region or were completely lost to follow-up (n = 206, 5%), or had demanded deletion of their address data (n = 12, 0.2%). Furthermore, 176 persons (4%) had died during follow-up time. Of the remaining 3,867 eligible persons, 174 could not be contacted, 218 were unable to come because they were too ill or had no time, and 395 were not willing to participate in this follow-up, resulting in a response rate of 79.6%. The current study was restricted to 2,970 subjects (1539 women and 1431 men) without missing values of any of the analytical variables (n = 110) (see Figure 1). The investigations were carried out in accordance with the Declaration of Helsinki, including written informed consent of all participants. All study methods were approved by the Ethics committee of the “Bayerische Landesärztekammer” Munich.

Known diabetes was defined as validated physician diagnosis or current use of antidiabetic agents. As type 2 diabetes mellitus constitutes approximately 95% of all cases of diabetes in the Western countries it can be assumed that most of the persons who have known diabetes in this study suffer from type 2 diabetes. After an overnight fast of at least 10 hours, all nondiabetic participants underwent a standard 75-g oral glucose tolerance test (OGTT) [14]. In persons with known diabetes no OGTT was carried out. NDD, i-IGT, i-IFG, and normal glucose tolerance (NGT) were defined according to the 1999 WHO diagnostic criteria based on both fasting and 2-h-glucose values [15]. We have used the original IFG criteria (6.1 to 6.9 mmol/l) for the present analysis, as recommended by the European Diabetes Epidemiology Group [16].

### Data collection

Information on sociodemographic variables, smoking habits, physical activity level, and alcohol consumption was gathered by trained medical staff during a standardized interview. Education was estimated by recording years of schooling completed. In addition all participants underwent an extensive standardized medical examination [17]. A regular smoker was defined as a subject who smoked at least one cigarette per day at baseline. Alcohol intake was categorized into two categories: men: <40 or ≥40 g/d; women: <20 or ≥20 g/d. Body mass index (BMI) was calculated as weight in kilograms divided by height in square meters. Waist circumference was measured at the level midway between the lower rib margin and the iliac crest. Systolic and diastolic blood pressure were measured three times at the right arm of seated subjects, after at least five minutes at rest, and by use of an oscillometric digital blood pressure monitor (HEM-705CP, Omron Corporation, Tokyo, Japan). The pause between readings was three minutes. The mean of the second and third measurement was calculated and used for the present analyses. Hypertension was defined as blood pressure values ≥140/90 mm Hg and/or use of antihypertensive medication given that the subjects were aware of being hypertensive. Individuals who participated in leisure time physical training during summer and winter and were active for at least one hour per week in either season were classified as being physically active.

### Clinical chemical measurements

A fasting venous blood sample was obtained from all study participants while sitting. Blood was collected without stasis cooled at 4–8°C and shipped on refrigerant packaging within 4 to 6 hours to the laboratory. Blood glucose was analyzed using a hexokinase method (GLU Flex, Dade Behring). Total serum cholesterol, and HDL cholesterol analyses were carried out using the CHOD-PAP method (CHOL Flex, and AHDL Flex). Triglycerides were measured with the GPO-PAP-method (TGL Flex, Dade Behring). Serum creatinine was measured using a modified kinetic Jaffé reaction. Serum concentrations of uric acid were measured on fresh samples by the uricase method (enzymatic color-test, URCA Flex, Dade Behring).

### Statistical analyses

Logistic regression models were used to test differences in prevalences and a general linear model (F-Test) was used to compare means (reference group NGT). The variables triglycerides and creatinine were markedly skewed and were therefore log-transformed in analyses where a normal distribution was required. We performed multinomial logistic regression analyses using the SAS procedure PROC LOGISTIC to study the association of uric acid with different categories of impaired glucose regulation. In these analyses the categorical dependent outcome had six groups (NGT, i-IFG, i-iGT, IFG/IGT, NDD, and known diabetes) and the reference group for comparison was NGT. In the analyses four models were fitted: The first model included uric acid, age (continuous) and sex. The second model included all previous factors plus hypertension (yes/no), physical activity (active/inactive), regular smoker (yes/no), high alcohol intake (yes/no), and serum creatinine (continuous). The third model included total cholesterol and HDL cholesterol (both continuous) in addition to all previous factors and the fourth model included BMI (continuous) in addition to all previous factors. The above analyses were repeated by including waist circumference (WC) as a continuous variable instead of BMI. Furthermore, the association between hyperuricemia and glucose intolerance categories was assessed using multinomial regression analysis. The cut-off points 7.0 mg/dl in men and 6.0 mg/dl in women were used in this analysis.The same stepwise modeling strategy (models 1–4) as described above was used in the multinomial analysis. Finally, in a logistic regression analysis the association between uric acid level (continuous) and all impaired glucose regulation groups combined as the outcome was investigated. In this analysis, study participants with i-IFG, i-IGT, IFG/IGT, NDD, and known diabetes were regarded as persons with impaired glucose regulation and persons with NGT built the reference group. All analyses were done for the total sample as well as separately for men and women. A p-value of <0.05 was considered statistically significant. All analyses were performed with SAS software version 9.1. (SAS Institute, Inc., Cary, NC, USA).

## Results

Sex-specific characteristics of the study sample according to glucose tolerance categories are shown in Table 1 (women) and Table 2 (men). Women and men with i-iFG, i-IGT, IFG/IGT, newly diagnosed diabetes, or known diabetes had higher uric acid levels than reference subjects with NGT. In both sexes glucose status categories also differed with respect to demographic and clinical factors. As compared to the NGT group, female and male subjects with impaired glucose status or newly diagnosed and known diabetes were older, more likely to be less educated, had a higher BMI or waist circumference, as well as a higher systolic blood pressure. Those women and men with impaired glucose status, newly diagnosed or known diabetes more often suffered from hypertension. Impaired glucose status and newly, as well as known diabetes were related to lower HDL cholesterol, higher triglycerides, higher creatinine values, higher alcohol intake (women only), non-smoking, and lack of physical activity. As compared to the NGT group, women and men with impaired glucose status or newly diagnosed diabetes had higher total cholesterol values, while persons with known diabetes had lower total cholesterol values.

**Table 1:** Study characteristics by NGT, i-IFG, i-IGT, IFG/IGT, NDD, and known diabetes mellitus, men (n = 1431) and women (n = 1539) aged 32–81 years, KORA F4 study.

	NGT	i-IFG	i-IGT	IFG/IGT	NDD	Known diabetes
Men	n = 971	n = 75	n = 147	n = 42	n = 61	n = 135
Women	n = 1,164	n = 38	n = 168	n = 27	n = 47	n = 95
**Age (years)**						
Men	52.8 (12.8)	60.8 (10.8)**	63.6 (11.2)**	63.1 (10.0)**	65.1 (10.4)**	68.2 (8.9)**
Women	52.8 (12.4)	62.3 (10.8)**	63.8 (11.5)**	66.3 (7.1)**	65.4 (10.6)**	66.3 (9.4)**
**Uric acid (mg/dL)**						
Men	5.8 (1.2)	6.7 (1.4)**	6.3 (1.2)**	6.4 (1.1)**	6.2 (1.5)*	6.3 (1.5)**
Women	4.3 (1.0)	5.2 (1.5)**	4.8 (1.1)**	5.5 (1.0)**	5.7 (1.5)**	5.6 (1.6)**
**Education (<12 years, %)**						
Men	47.0	50.7	53.1	47.6	52.5	67.4**
Women	63.8	65.8	78.6**	77.8	76.6	80.0**
**BMI (kg/m^2^)**						
Men	27.0 (3.7)	29.7 (3.1)**	29.5 (4.8)**	30.5 (5.1)**	30.8 (4.6)**	29.9 (4.6)**
Women	26.2 (4.8)	30.2 (6.7)**	29.4 (4.8)**	31.3 (4.9)**	31.5 (4.1)**	33.1 (6.0)**
**Waist circumference (cm)**						
Men	96.9 (10.6)	104.9 (10.8)**	104.8 (14.1)**	107.0 (13.4)**	107.9 (11.5)**	107.1 (11.7)**
Women	85.1 (11.9)	97.5 (14.1)**	93.3 (12.1)**	101.6 (12.9)**	100.1 (9.1)**	102.5 (13.6)**
**Systolic blood pressure (mm Hg)**						
Men	125.0 (16.3)	132.1 (18.0)**	131.9 (18.1)**	135.8 (18.2)**	135.1 (18.4)**	134.8 (20.0)**
Women	114.2 (16.8)	128.2 (22.0)**	121.6 (19.0)**	131.7 (13.7)**	127.1 (15.5)**	129.8 (19.2)**
**Diastolic blood pressure (mm Hg)**						
Men	77.3 (9.7)	79.0 (10.0)	78.2 (11.3)	82.4 (10.2)**	79.4 (11.5)	75.6 (10.6)
Women	72.6 (9.3)	74.4 (8.9)	73.1 (9.3)	78.7 (7.3)**	74.0 (9.9)	73.0 (10.2)
**Actual hypertension (%)**						
Men	31.5	56.0**	65.3**	78.6**	75.4**	78.5**
Women	24.2	50.0**	45.8**	55.6**	80.9**	81.1**
**Total cholesterol (mg/dL)**						
Men	213.4 (36.9)	219.2 (44.9)	221.3 (42.6)*	223.3 (37.6)	216.7 (44.7)	194.0 (37.5)**
Women	216.1 (39.2)	231.7 (36.6)*	228.1 (42.3)**	233.5 (37.5)*	222.8 (39.4)	214.2 (40.2)
**HDL cholesterol (mg/dL)**						
Men	51.4 (12.1)	48.7 (13.6)	50.2 (13.7)	47.3 (13.2)*	46.5 (12.3)**	46.3 (10.7)**
Women	62.8 (14.1)	55.2 (12.6)**	59.7 (13.5)**	53.0 (11.7)**	50.5 (12.5)**	52.8 (11.2)**
**Triglycerides (mg/dL)** §						
Men	110.0 (1.7)	140.7 (1.7)**	136.9 (1.7)**	162.1 (1.9)**	156.5 (1.9)**	136.9 (1.7)**
Women	85.1 (1.6)	125.9 (1.6)**	110.1 (1.5)**	154.7 (1.4)**	155.4 (1.7)**	139.1 (1.8)**
**Creatinine (mg/dL)** §						
Men	0.98 (1.18)	0.99 (1.21)	1.01 (1.21)	1.0 (1.17)	1.03 (1.25)	1.07 (1.31)**
Women	0.78 (1.18)	0.79 (1.20)	0.81 (1.21)*	0.87 (1.25)**	0.85 (1.20)**	0.86 (1.27)**
**Alcohol intake (%)**						
Men ≥40.0 g/d	19.4	24.0	20.4	31.0	27.9	19.3
Women ≥20.0 g/d	15.6	23.7	11.9	14.8	14.9	8.4
**Regular smoking (%)**						
Men	20.5	8.0**	8.8**	9.5	14.8	12.6*
Women	15.2	13.2	4.8**	7.4	6.4	7.4*
**Physically active during leisure time (%)**						
Men	57.9	50.7**	45.6	50.0	52.5	34.8**
Women	58.5	34.2**	53.0	59.3	40.4*	39.0**

NGT, normal glucose tolerance; NDD, newly detected diabetes; i-IFG, isolated impaired fasting glucose; i-IGT, isolated impaired glucose tolerance.

§ geometric mean (SD);

* p<.05;

** p<.01 for comparisons with NGT.

**Table 2:** Association of uric acid values with i-IFG, i-IGT, IFG/IGT, NDD, and known diabetes (comparisons with NGT), KORA F4 study.

	i-IFG	i-IGT	IFG/IGT	NDD	Known diabetes
Total sample	(n = 113)	(n = 315)	(n = 69)	(n = 108)	(n = 230)
Men	(n = 75)	(n = 147)	(n = 42)	(n = 61)	(n = 135)
Women	(n = 38)	(n = 168)	(n = 27)	(n = 47)	(n = 95)
† **Model 1: OR (95%-CI)**					
Total sample	1.66 (1.43–1.92)	1.26 (1.14–1.40)	1.57 (1.31–1.89)	1.57 (1.35–1.83)	1.55 (1.38–1.73)
Men	1.59 (1.33–1.90)	1.24 (1.08–1.43)	1.35 (1.06–1.71)	1.20 (0.98–1.47)	1.29 (1.11–1.50)
Women	1.77 (1.36–2.31)	1.31 (1.12–1.54)	1.99 (1.49–2.67)	2.24 (1.79–2.80)	2.07 (1.73–2.48)
‡ **Model 2: OR (95%-CI)**					
Total sample	1.73 (1.47–2.03)	1.25 (1.12–1.40)	1.49 (1.22–1.83)	1.45 (1.23–1.71)	1.40 (1.24–1.59)
Men	1.67 (1.37–2.04)	2.21 (1.04–1.41)	1.26 (0.97–1.63)	1.09 (0.88–1.36)	1.19 (1.02–1.40)
Women	1.90 (1.41–2.55)	1.32 (1.12–1.57)	1.95 (1.40–2.72)	2.11 (1.63–2.72)	1.86 (1.52–2.28)
§ **Model 3: OR (95%-CI)**					
Total sample	1.61 (1.37–1.91)	1.20 (1.07–1.34)	1.33 (1.09–1.64)	1.30 (1.09–1.53)	1.30 (1.15–1.48)
Men	1.59 (1.29–1.96)	1.17 (1.00–1.37)	1.15 (0.88–1.50)	0.99 (0.79–1.24)	1.12 (0.95–1.32)
Women	1.71 (1.25–2.32)	1.25 (1.05–1.49)	1.69 (1.20–2.39)	1.84 (1.41–2.39)	1.69 (1.37–2.09)
**||Model 4: OR (95%-CI)**					
Total sample	1.50 (1.26–1.77)	1.10 (0.98–1.24)	1.22 (0.98–1.50)	1.18 (1.00–1.40)	1.17 (1.03–1.34)
Men	1.49 (1.21–1.84)	1.09 (0.93–1.28)	1.06 (0.81–1.39)	0.91 (0.72–1.14)	1.04 (0.88–1.23)
Women	1.57 (1.15–2.14)	1.14 (0.95–1.36)	1.52 (1.07–2.16)	1.67 (1.28–2.17)	1.47 (1.18–1.82)
P-value for uric acid*sex interaction (Total sample)	0.8027	0.7449	0.0720	0.0007	0.0172

Odds ratio (OR) expressed per mg/dL increment in uric acid concentration; if the confidence intervals around the odds ratios overlap, then the odds ratios are not significantly different

† Model 1: adjusted for age and sex (only total sample).

‡ Model 2: adjusted for age, sex, actual hypertension, regular smoking, physical activity, alcohol intake, and creatinine.

§ Model 3: adjusted for age, sex, actual hypertension, regular smoking, physical activity, alcohol intake, creatinine, total cholesterol, and HDL cholesterol.

|| Model 4: adjusted for age, sex, actual hypertension, regular smoking, physical activity, alcohol intake, creatinine, total cholesterol, HDL cholesterol, and BMI.

The results of the multinomial regression analysis are given in Table 2. In the total sample uric acid concentration were significantly associated with glucose status groups after age- and sex- as well as after multivariable adjustment. In the age- and sex-adjusted model the OR per mg/dL increment of uric acid was 1.66 (95% CI 1.43–1.92) for i-IFG, 1.26 (95% CI 1.14–1.40) for i-IGT, 1.57 (95% CI 1.31–1.89) for IFG/IGT, 1.57 (95% CI 1.35–1.83) for NDD and 1.55 (95% CI 1.38–1.73) for known diabetes. Further adjustment for smoking status, alcohol intake, physical activity, actual hypertension, total cholesterol, HDL cholesterol and creatinine values somewhat attenuated the associations, but the ORs per mg/dL uric acid increment were still significantly increased for all glucose status groups compared to persons with NGT. Additional adjustment for BMI further attenuated the findings and the associations between uric acid level and the categories i-IGT and IFG/IGT lost significance, while the association between uric acid level and i-IFG, NDD, and known diabetes remained significant (the OR per mg/dL increment of uric acid was 1.50 for i-IFG, 1.10 for i-IGT, 1.22 for IFG/IGT, 1.18 for NDD, and 1.17 for known diabetes). There were significant interactions between sex and uric acid levels (see Table 2). As shown in Table 2 uric acid levels were more strongly associated with the different glucose tolerance categories in women compared with men. In women after multivariable adjustment the associations remained statistically significant for i-IFG, IFG/IGT, NDD, and known diabetes, but lost significance for i-IGT (model 4: i-IFG: OR 1.57, i-IGT: OR 1.14, IFG/IGT: OR 1.52, NDD: OR 1.67, known diabetes: OR 1.47). However, in men, in multivariable analyses there was only a significant association between uric acid levels and i-IFG, all other associations were non-significant (model 4: i-IFG: OR 1.49, i-IGT: OR 1.09, IFG/IGT: OR 1.06, NDD: OR 0.91, known diabetes: OR 1.04).

The strength of the relationship between uric acid levels and glucose status groups was unchanged after adjusting for waist circumference instead of BMI in the different models (data not shown).

When performing the multinomial regression analysis with a categorized variable hyperuricemia (cut-off value of 6.0 mg/dl for women and 7.0 mg/dl for men) the results found in the analysis using the continuous uric acid variable were confirmed. Altogether, 286 men and 157 women suffered from hyperuricemia.Hyperuricemia was more strongly associated with the different glucose tolerance categories in women compared with men. In women after multivariable adjustment the associations remained statistically significant for all single categories of glucose intolerance (model 4: i-IFG: OR 1.57 (95% CI 1.80–10.41), i-IGT: OR 1.89 (95% CI 1.10–3.24), IFG/IGT: OR 2.88 (95% CI 1.11–7.49), NDD: OR 3.26 (95% CI 1.53–6.92), known diabetes: OR 2.33 (95% CI 1.26–4.31). In men, in multivariable analyses there was only a significant association between hyperuricemia and i-IFG, all other associations were non-significant (model 4: i-IFG: OR 2.48 (95% CI 1.44–4.26), i-IGT: OR 1.13 (95% CI 0.71–1.78), IFG/IGT: OR 1.15 (95% CI 0.55–2.42), NDD: OR 0.94 (95% CI 0.48–1.81), known diabetes: OR 1.11 (95% CI 0.68–1.82).

In the analysis regarding the association between uric acid level (continuous) and impaired glucose regulation groups combined (reference group NGT) as the outcome, it could be shown, that in women after multivariable adjustment uric acid levels were significantly associated with impaired glucose regulation (the OR per mg/dL increment of uric acid was 1.34; 95% CI 1.17–1.54); in men, however, the association was not significant (OR per mg/dl increment of uric acid: 1.11; 95% CI 0.99–1.24).

## Discussion

Uric acid levels were significantly associated with different categories of impaired glucose regulation independent of known metabolic risk factors and lifestyle variables in men and women from the general population. The associations were more pronounced in women than in men. It could be shown that prediabetic subjects and persons with newly diagnosed diabetes had higher uric acid levels than normoglycemic subjects. In the present investigation persons with known diabetes had also higher uric acid levels than non-diabetic persons, a finding which is contrary to other studies [11]–[13], [18].

Prior cohort studies have reported gender differences in the relationship between uric acid levels and the development of diabetes [17]–[20]. An earlier study of a different sample of the MONICA/KORA study already demonstrated a clear association between uric acid levels and incident diabetes that was present in women and not in men [17]. In another study, Yamada et al. reported that elevated serum uric acid predicted IFG and type 2 diabetes only in Japanese women but not in men undergoing health checkups [20]. Chou et al found that serum uric acid levels were associated with insulin resistance and plasma glucose levels more strongly in women than in men [19].

Only a few, very early, cross-sectional studies have investigated the association between uric acid levels and different glucose tolerance groups in men and women from the general population [11]–[13]. It was found that uric acid was significantly elevated in people with IGT [11], and in those with fasting plasma glucose up to 7.0–8.0 mmol/l in men and to 9.0 mmol/l in women [12], [21], [22]. Among persons with NDD notably low uric acid levels were observed [11]–[13], [21], a finding which is in contrast with the present results.

In the present study in multivariable adjusted multinomial regression analysis we could show that among women high uric acid levels were associated with each single category of glucose intolerance (besides i-IGT). However, this was not the case in men, in whom only the association between uric acid levels and i-IFG persisted after multivariable adjustment. These findings underscore a possible role of uric acid in the early pathogenesis of type 2 diabetes, particularly in women. It seems that hyperuricemia had a high specificity for men and women with i-IFG, a state of impaired glucose regulation, which differs from other prediabetic states [23]. Although both types of prediabetes that is i-IFG and/or i-IGT are associated with an increased risk for developing type 2 diabetes mellitus, they manifest distinct metabolic abnormalities. Impaired fasting glucose is more strongly associated with insulin resistance than impaired glucose tolerance and the aetiologies of i-IFG and i-IGT seem to differ [23]. Thus, one possible explanation for the found differences in the present work is that uric acid may play a main role in the aetiology of insulin resistance. However, the underlying pathophysiologic mechanisms for the strong association between hyperuricaemia and i-IFG and the reasons for the gender differences are not clear and should be subject of further investigations. It has been shown, that there are strong sex-specific effects in the genetic basis of uric acid production [24], possibly suggesting a genetic basis for the sex differences even in glucose metabolism.

Thus, although it remains unclear whether or not hyperuricemia is a consequence of insulin resistance or its precursor [25], [26] and the results of a very recent study do not support a causal role for serum uric acid in the development of type 2 diabetes [27], the simple measurement of uric acid might help to identify persons with impaired glucose regulation, in particular persons with i-IFG. An early identification of subjects suffering from a prediabetic state would help to prevent or delay the development of diabetes and its complications.

A number of mechanisms have been suggested by which uric acid could affect changes in glucose metabolism. Elevated serum uric acid was found to be associated with oxidative stress and systemic inflammation both of which play crucial roles in the development of diabetes mellitus [28]. Furthermore, uric acid decreases endothelial nitric oxide production and thus induces endothelial dysfunction and insulin resistance [29], [30]. Finally, uric acid is related to increased renal glomerular pressure and increased renal sodium reabsorption, and these renal reactions are greatly enhanced by high insulin concentrations [25]. The combined effects of insulin resistance and high uric acid levels on renal functions may contribute to increased glucose intolerance, hypertension, and the development of diabetes.

The cross-sectional design of the study represents a limitation, implicating that cause and effect relationships cannot be discerned. Furthermore, although we adjusted for a variety of important confounding variables, such as alcohol consumption, smoking, and renal function, we cannot exclude that unknown risk factors may have biased or confounded the present analysis. The strength of the study is the large number of subjects randomly drawn from the general population, and the availability of data on lifestyle and multiple metabolic risk factors.

In conclusion, serum concentrations of uric acid were independently associated with different states of impaired glucose regulation in particular in women from the general population. Further studies, in particular prospective studies are needed to investigate the contribution of uric acid to the pathogenesis of prediabetic states and finally to the manifestation of type 2 diabetes.
